# mBatchNet: an interactive web server for diagnosis, correction, and benchmarking of batch effects in microbiome data

**DOI:** 10.1093/bioinformatics/btag538

**Published:** 2026-07-21

**Authors:** Chentong Sun, Shiyuan Wang, Qiwei Zhang, Ruishan Liu, Yuxuan Du

**Affiliations:** Department of Electrical Engineering, The University of Texas at San Antonio, San Antonio, TX 78249, United States; College of Engineering and Applied Science, University of Colorado Boulder, Boulder, CO 80309, United States; Department of Electrical Engineering, The University of Texas at San Antonio, San Antonio, TX 78249, United States; Department of Computer Science, University of Southern California, Los Angeles, CA 90089, United States; Department of Computer Science, University of Southern California, Los Angeles, CA 90089, United States; Department of Electrical Engineering, The University of Texas at San Antonio, San Antonio, TX 78249, United States

## Abstract

**Summary:**

Batch-effect diagnosis and correction are important for reproducible microbiome analysis and cross-study integration. Several batch-correction algorithms are available, but applying and comparing established methods in practice remains nontrivial because they differ in assumptions, accepted inputs, parameters, and evaluation outputs. Here, we present mBatchNet, an interactive web server for applying established batch-correction methods to processed microbiome feature tables and evaluating their effects within a single workflow. The server supports correction methods spanning recent microbiome-oriented approaches and established general-purpose baselines, validates uploaded feature tables and metadata, flags batch-target association, applies matched pre- and post-correction diagnostics, and exports corrected matrices, statistical summaries, run logs, and reproducibility records. In a 16S ribosomal RNA (rRNA) anaerobic digestion case study, mBatchNet revealed method-dependent differences in batch attenuation and phenotype preservation, highlighting its utility for comparing correction strategies.

**Availability and implementation:**

mBatchNet is freely available without login at https://mbatchnet.com/. The latest source code is available at https://github.com/gilmore307/mBatchNet, and is archived at https://doi.org/10.5281/zenodo.20767444. The server is implemented with a Python/Dash front end and coordinated Python/R back-end analysis scripts.

## 1 Introduction

High-throughput microbiome studies are increasingly integrated across cohorts, centres, and time points, but abundance profiles are highly susceptible to batch effects arising from sample handling, DNA extraction, library preparation, sequencing runs, and preprocessing pipelines (Sinha *et al.* 2017, McLaren *et al.* 2019). Microbiome feature tables add practical complexity because they are sparse, compositional, and often zero-inflated. Consequently, batch-correction methods designed for microbiome data may differ in accepted input scale, zero handling, covariate adjustment, and modelling assumptions (Ling *et al.* 2022, Ma *et al.* 2022, Wang and Lê Cao 2023, Austin *et al.* 2025, Yuan and Wang 2025). Recent microbiome-oriented batch-effect correction methods, including ConQuR (Ling *et al.* 2022), MMUPHin (Ma *et al.* 2022), PLSDA-batch (Wang and Lê Cao 2023), DEBIAS-M (Austin *et al.* 2025), and MetaDICT (Yuan and Wang 2025), address these data characteristics using complementary assumptions. For applied users, the practical challenge is workflow integration across heterogeneous method implementations. Specifically, methods may expect different input representations, expose different parameter sets, use covariates differently, and generate method-specific outputs, making side-by-side correction evaluation and reproducible reporting laborious when tools are run separately. Existing resources have important but more limited scopes for current microbiome batch-correction workflows. BatchServer (Zhu *et al.* 2021) is a general-omics web server centred on ComBat-based correction (Johnson *et al.* 2007) with a focused diagnostic set, whereas MBECS provides microbiome correction and evaluation routines for R users (Olbrich *et al.* 2023) but is centred on earlier established correction approaches. Neither resource provides a microbiome-focused browser workflow that combines recent and established correction methods with processed feature-table validation, metadata mapping, batch-target association screening, method guidance, matched pre- and post-correction diagnostics, output navigation, and reproducibility records within one session.

We developed mBatchNet as a workflow-focused web-server resource for executing established correction methods, reviewing batch-target metadata structure, benchmarking correction outcomes, and exporting corrected matrices and figures within a reproducible session. The public deployment includes loadable example data, help pages, and a tutorial so that users can reproduce the complete analysis from data upload to result interpretation.

## 2 Features and implementation

mBatchNet is implemented with a Python/Dash web interface and coordinated Python/R back-end routines. The interface is organized into four user-facing pages: Upload Files, Pre-correction Assessment, Batch Effect Correction, and Post-correction Assessment, which match the analysis workflow. The Upload Files page validates the processed feature table and metadata, maps metadata columns to analysis roles representing batch, target/phenotype, and optional covariates, and screens batch-target association before correction. The Pre-correction Assessment and Post-correction Assessment pages apply matched diagnostics before and after correction, and the Batch Effect Correction page runs selected methods while recording method-specific parameters and logs.

The server accepts processed sample-by-feature microbiome comma-separated value (CSV) tables with matching sample metadata. Because analysis begins with feature tables rather than raw reads, the same workflow can be used for 16S-derived operational taxonomic unit (OTU) and amplicon sequence variant (ASV) tables and shotgun metagenomic taxonomic or functional abundance/count profiles after upstream profiling. Raw sequencing files such as FASTQ are not accepted. To protect public-server availability and provide clear expectations before job submission, the current deployment accepts feature-table CS5 files with up to 500 samples, 1000 features, and 10 MB per file. Metadata CSV files are also limited to 10 MB and five columns, including batch labels, target/phenotype labels, and optional covariates. Users with larger datasets should use the local installation/source-code version rather than the public web server. During file upload, the server performs pre-run validation before correction starts. Inputs are rejected if they exceed the public-server limits or fail required format checks, including missing or invalid numeric values, all-zero sample rows, metadata mismatches, missing required metadata columns, or invalid batch/target selections. Advisory warnings are displayed for inputs that can still be analysed but may require user review, including all-zero feature columns, outlier flags, and strong batch-target association. The batch-target association warning gives users an early heads-up that correction results may require cautious interpretation when technical batch labels and biological target/phenotype labels are strongly associated. All blocking errors and advisory warnings are shown in the web interface and saved in the downloadable validation report. Detailed public-server limits, block checks, and advisory warnings are provided in Table S1.

The Batch Effect Correction page currently includes 12 correction methods spanning recent microbiome-oriented approaches and general-purpose baselines. Each method is presented in an expandable panel that includes a plain-language method description, package/source and citation/reference links, and exposed-parameter explanations with default values where applicable. In this paper, the illustrative case study uses five representative microbiome-oriented methods, namely ConQuR (Ling *et al.* 2022), MMUPHin (Ma *et al.* 2022), PLSDA-batch (Wang and Lê Cao 2023), DEBIAS-M (Austin *et al.* 2025), and MetaDICT (Yuan and Wang 2025), whereas the complete supported-method list and practical guidance are summarized in Supplementary Note 1, Table S2, and on the Help page of the web server. To provide runtime expectations, the Batch Effect Correction page displays reference elapsed times and run-specific elapsed times for each method, and each downloadable output bundle includes a runtime summary. Table S3 reports elapsed time and peak memory measured on the bundled example dataset used in the manuscript case study under default parameters. These example-based estimates are not worst-case guarantees, because runtime and memory depend on sample number, feature number, sparsity, selected method, parameter settings, and server load. Users with datasets exceeding the public-server limits are advised to use the local/source-code installation rather than the public web server.

The assessment pages apply the same metrics before and after correction so that outputs from different methods can be interpreted under a common diagnostic framework. Diagnostics are organized into four complementary categories: ordination and embedding analyses [principal component analysis (PCA), principal coordinates analysis (PCoA), and non-metric multidimensional scaling (NMDS)] for dominant sample structure; distance-matrix tests [analysis of similarities (ANOSIM) and permutational multivariate analysis of variance (PERMANOVA)] for residual batch-associated separation; variance-attribution analyses [feature-wise analysis of variance (ANOVA), partial redundancy analysis (pRDA), and principal variance component analysis (PVCA)] for batch, target, overlap, and residual components; and neighbourhood or label-preservation metrics [*k*-nearest-neighbour (*k*-NN) alignment, entropy of batch mixing, and silhouette summaries] for local cross-batch mixing and target separation. Distance-based diagnostics use Aitchison and Bray–Curtis dissimilarities for complementary purposes. Aitchison distance is computed as the Euclidean distance between centred log-ratio (CLR)-transformed profiles and compares samples in log-ratio space, making it suitable for compositional ordination and Aitchison-based batch-association diagnostics (Aitchison 1982). Bray–Curtis dissimilarity is computed after total-sum scaling each sample to a non-negative relative-abundance profile, so it summarizes community-composition differences rather than total library-size differences (Bray and Curtis 1957). Detailed descriptions of the diagnostic metrics used to evaluate correction outputs, together with the sample-wise zero-replacement rule applied before CLR transformation, are provided in Supplementary Note 2.

Finally, to support reproducibility and output navigation, each downloadable session bundle includes corrected matrices, diagnostic figures, statistical summaries, output and parameter manifests, session-configuration files, validation and runtime reports, execution logs, and an exported shell script (execution_commands.sh). The exported script records the server-side preprocessing and correction-method commands generated from the user-selected methods and parameters, including method-specific flags where available. The output manifest (output_summary.json) describes the generated files so that users can quickly locate method-specific corrected tables, diagnostic figures, statistical summaries, and logs. The parameter manifest, validation report, runtime summary, and execution logs record the selected correction methods, metadata mapping, method-specific parameters, input checks, elapsed-time information, and run status for reproducibility and troubleshooting. The main output and reproducibility records are summarized in Table S4.

## 3 Results

### 3.1 Illustrative case study

We demonstrated mBatchNet using the anaerobic digestion 16S ribosomal RNA (rRNA) dataset from a phenol-perturbation experiment, following the benchmark setup of PLSDA-batch (Chapleur *et al.* 2016, Wang and Lê Cao 2023). This example is bundled in the server so that users can reproduce the analysis directly from the web interface. The dataset comprises 231 operational taxonomic units (OTUs) measured across 75 samples. The target/phenotype label was initial phenol concentration (0–0.5 versus 1–2), the batch label represented five processing dates, and treatment duration was included as an optional covariate where supported. The two target groups contained 26 and 49 samples, and batch sizes ranged from nine to 21 samples. After data upload, mBatchNet generates a mosaic plot summarizing the joint distribution of samples across batch and phenotype groups. The resulting plot for this dataset is shown in Fig. S1.

**Figure 1:**
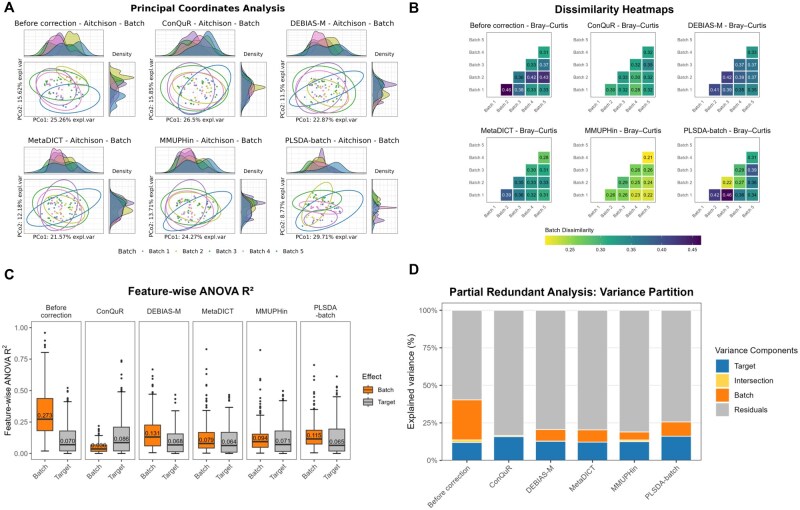
Representative mBatchNet outputs for the anaerobic digestion example. (A) Aitchison PCoA plots coloured by batch. (B) Bray–Curtis batch-dissimilarity summaries across correction methods. (C) Per-feature ANOVA $R^{2}$ values for batch and phenotype. (D) pRDA variance partitioning into phenotype, batch, shared, and residual components. Complete numerical summaries for the main and supplementary diagnostic figures are provided in Supplementary Tables S5–S9..

The illustrative analysis uses five representative microbiome-oriented methods (ConQuR, MMUPHin, PLSDA-batch, DEBIAS-M, and MetaDICT) to show how mBatchNet compares correction outputs under a common diagnostic workflow. To keep the main figure readable within the page limit, Fig. 1 focuses on these five methods. All 12 supported methods are documented in Table S2, and compact all-method case-study results are provided in Supplementary Tables S5–S9. Pre-correction diagnostics showed clear batch-associated structure (Fig. 1). The diagnostic panels were interpreted as complementary evidence rather than as a single method ranking and are linked to the metric definitions in Supplementary Note 2. Figure S1 provides sample-composition context by showing sample composition across batch and target groups. Figure 1A shows global batch-associated separation and mixing in Aitchison ordination space; Fig. 1B shows Bray–Curtis batch-dissimilarity summaries; Fig. 1C compares feature-level batch and target/phenotype associations; and Fig. 1D partitions multivariate variation into target, batch, shared, and residual components by pRDA. Supplementary Figures S2–S6 provide additional target- and batch-coloured ordinations, whereas Supplementary Figs. S7 and S8 provide PVCA and neighbourhood/embedding metrics for cross-checking variance attribution, local batch mixing, and target separation. Full numerical summaries corresponding to the main and supplementary diagnostic figures are provided in Supplementary Tables S5–S9. In Aitchison ordinations, the uncorrected data clustered by processing date (Fig. 1A; Table S5), and Bray–Curtis distance-based analyses indicated strong batch association (Fig. 1B; Table S6; ANOSIM R=0.637, permutation *P* = .001; PERMANOVA $R^{2}=0.401$, permutation *P* = .001). Among the corrected outputs, ConQuR, DEBIAS-M, and MMUPHin had Bray–Curtis ANOSIM values close to zero or below zero, and their PERMANOVA $R^{2}$ values were lower than the uncorrected value, although MMUPHin and DEBIAS-M retained statistically detectable residual batch association by PERMANOVA. MetaDICT also reduced the ANOSIM statistic (R=0.051, permutation *P* = .051) while retaining detectable PERMANOVA association ($R^{2}=0.128$, permutation *P* = .002). PLSDA-batch retained stronger residual batch separation by ANOSIM (R=0.377, permutation *P* = .001), with PERMANOVA $R^{2}=0.098$ and permutation *P* = .013. Thus, outputs were interpreted across panels rather than by one score: reduced batch separation supported batch attenuation, whereas retained target/phenotype structure supported phenotype preservation. This case study illustrates the main role of mBatchNet, namely to provide a harmonized environment in which users can inspect batch-target metadata structure, compare multiple correction outputs, and interpret correction trade-offs using consistent visual and statistical evidence. These observations are dataset-specific and are intended to illustrate how mBatchNet supports evidence-based method comparison rather than to rank correction methods universally or recommend one method for all microbiome datasets.

## 4 Conclusion and discussion

mBatchNet provides an accessible and reproducible web-based framework for diagnosing, correcting and benchmarking batch effects in processed microbiome feature tables. Rather than prescribing a universal best method, mBatchNet encourages users to run multiple plausible correction methods instead of relying on one default method, and to compare their outputs using the same pre- and post-correction diagnostics. In practice, users should look for methods that reduce batch-associated structure while preserving target/phenotype-associated structure, and should interpret disagreements among ordination, distance-based tests, variance-attribution summaries, neighbourhood metrics, and runtime estimates as evidence of method-dependent trade-offs rather than as a single automatic ranking. By integrating established correction methods with harmonized diagnostics, input validation, example data, method guidance, and downloadable reproducibility records, the server supports transparent microbiome data integration and downstream analysis. Future releases will explore extending the current validation and advisory-warning framework with optional, user-controlled preprocessing and quality-control utilities, such as explicit missing-value handling and configurable sample- or feature-filtering decisions, with user-selected actions recorded in the downloadable reproducibility records.

## Supplementary Material

btag538_Supplementary_Data

## Data Availability

The public mBatchNet web server is hosted at https://mbatchnet.com/. The site is free and open to all users and does not require login. The source code is available at https://github.com/gilmore307/mBatchNet, and is archived at https://doi.org/10.5281/zenodo.20767444. The anaerobic digestion 16S rRNA amplicon datasetunderlying this article is available in the PLSDA-batch repository (Wang and Lê Cao 2023) at https://github.com/EvaYiwenWang/PLSDAbatch/.
