# Supplementary material for: mBatchNet: an interactive web server for diagnosis, correction, and benchmarking of batch effects in microbiome data
Source: Bioinformatics. 2026 Jul 21;42(7):btag538. doi: 10.1093/bioinformatics/btag538 (PMC13423235; doi:10.1093/bioinformatics/btag538)
Supplement: btag538_Supplementary_Data [file btag538_supplementary_data.pdf]

# Supplementary Information

mBatchNet: an interactive web server for diagnosis, correction, and benchmarking of batch effects in microbiome data

Chentong Sun[1,2], Shiyuan Wang[1], Qiwei Zhang[1], Ruishan Liu[3] and Yuxuan Du*[1]

[1]Department of Electrical Engineering, The University of Texas at San Antonio, San Antonio, TX, 78249, USA
[2]College of Engineering and Applied Science, University of Colorado Boulder, Boulder, CO, 80309, USA
[3]Department of Computer Science, University of Southern California, Los Angeles, CA, 90089, USA

## Table of contents

**Supplementary Notes** ........ 2

**Supplementary Tables** ........ 4

**Supplementary Figures** ........ 13

**Supplementary References** ........ 21

*Corresponding author. Email: yuxuan.du@utsa.edu

# Supplementary Notes

## Supplementary Note 1. Supported correction methods and case-study method scope

mBatchNet currently supports 12 correction methods, spanning recent microbiome-oriented approaches and general-purpose baselines (Supplementary Table S2). In the web server, the Batch Effect Correction page provides expandable method explanations with package/source and citation/reference links, and the Config controls expose adjustable method parameters with default values and tooltips where applicable. The Help page mirrors these method descriptions and parameter explanations, while Supplementary Table S2 summarizes practical guidance for all supported methods. For the illustrative manuscript case study, we selected five representative microbiome-oriented methods–ConQuR (Ling et al., 2022), MMUPHin (Ma et al., 2022), PLSDA-batch (Wang and Lê Cao, 2023), DEBIAS-M (Austin et al., 2025), and MetaDICT (Yuan and Wang, 2025)–to show how mBatchNet compares correction outputs under a common diagnostic workflow. The remaining supported methods are documented on the server and in Supplementary Table S2 so that users can identify plausible methods for their analysis goal. Supplementary Tables S5–S9 provide a compact all-method server-run summary for the bundled anaerobic digestion dataset. These tables are illustrative and should not be interpreted as an exhaustive benchmark or universal ranking. They provide the numerical counterparts to the main and supplementary diagnostic figures, including distance-based batch-association tests in Aitchison and Bray–Curtis spaces, feature-wise ANOVA, pRDA/PVCA variance-partitioning summaries, neighbourhood or target-preservation metrics, and runtime. Permutation $p$-values for the distance-based analyses were computed using 999 permutations from the uncorrected and corrected output matrices.

## Supplementary Note 2. Transformations and diagnostic metrics

**CLR transformation and Aitchison distance.** For centred log-ratio (CLR) transformation, count or abundance matrices are first converted to relative-abundance profiles by total-sum scaling. Within each sample, zero entries are replaced before log transformation by a small positive value equal to the larger of $10^{-6}$ and 65% of the smallest positive relative abundance in that sample. If a sample is all zero, all entries are replaced by $10^{-6}$ as a defensive fallback; however, user-uploaded data with all-zero sample rows are blocked during validation. The log-transformed profile is then centred by subtracting the sample-wise mean log abundance. Aitchison distance is computed as the Euclidean distance between CLR-transformed profiles and is used for compositional ordination and downstream diagnostics.

**Bray–Curtis dissimilarity.** Bray–Curtis analyses operate on non-negative relative-abundance profiles. In practical terms, each sample is total-sum scaled before Bray–Curtis dissimilarities are computed, so the resulting dissimilarity summarizes differences in community composition rather than differences in total library size. Bray–Curtis dissimilarity is not computed on CLR-transformed data. All-zero sample rows and non-numeric values are rejected during validation before Bray–Curtis analyses are run.

**Ordination diagnostics.** The server visualizes dominant structure using principal component analysis (PCA) in CLR/Aitchison space and principal coordinates analysis (PCoA) and non-metric multidimensional scaling (NMDS) on Aitchison and Bray–Curtis dissimilarities. Plots are coloured by batch and by target label to inspect batch mixing and phenotype separation. For NMDS, the

server reports the stress value, a goodness-of-fit measure for low-dimensional representation, where lower stress indicates a more faithful embedding.

**Distance-matrix tests and dissimilarity summaries.** Residual batch association is quantified using analysis of similarities (ANOSIM) and permutational multivariate analysis of variance (PERMANOVA) on Aitchison and/or Bray–Curtis distances (Bray and Curtis, 1957; Li et al., 2019). ANOSIM reports a separation statistic $R$, where larger $R$ indicates stronger between-group separation (Clarke, 1993). PERMANOVA reports an effect size $R^2$, corresponding to the fraction of distance variation explained by the tested factor (Anderson, 2001). Dissimilarity heatmaps summarize mean between-batch distances across batch pairs; more homogeneous values indicate weaker batch-driven separation.

**Variance-partitioning analyses.** To quantify how compositional variation is apportioned among target, batch, and their overlap, mBatchNet reports partial redundancy analysis (pRDA) and principal variance component analysis (PVCA) under Aitchison geometry (Wang and Lê Cao, 2023). pRDA partitions variance into target, batch, intersection, and residual components. The intersection term reflects overlap between batch-associated and target-associated structure, with larger values indicating stronger shared technical and biological variation. PVCA yields an analogous target/batch/intersection/residual decomposition by applying a variance-components model to retained principal component scores and aggregating components across principal components using eigenvalue-based weights. mBatchNet also computes per-feature one-way analysis of variance (ANOVA) $R^2$ values for batch and target.

**Neighbourhood and embedding metrics.** Local mixing is quantified using a $k$-nearest-neighbour ($k$-NN) alignment score, where higher values indicate stronger cross-batch neighbourhood mixing (Butler et al., 2018; Luecken et al., 2022). mBatchNet also computes a Uniform Manifold Approximation and Projection (UMAP) embedding (McInnes et al., 2018) and reports an entropy-of-batch-mixing score on UMAP neighbourhoods, where higher values indicate more uniform batch mixing (Haghverdi et al., 2018; Luecken et al., 2022). To quantify phenotype preservation and separation, the server reports the silhouette coefficient with respect to target labels on the UMAP coordinates, where higher values indicate stronger target separation (Rousseeuw, 1987).

**Connection to case-study figures.** In the anaerobic digestion example, Supplementary Figure S1 summarizes the sample composition across batch and target groups and provides metadata-distribution context before correction. Figure 1A in the main text and Supplementary Figures S2–S6 show ordination-based diagnostics for global batch mixing and target/phenotype separation. Figure 1B in the main text summarizes Bray–Curtis dissimilarities and distance-based batch-association tests. Figure 1C in the main text shows feature-wise ANOVA summaries for batch and target/phenotype associations, and Figure 1D in the main text and Supplementary Figure S7 show pRDA and PVCA variance-partitioning summaries. Supplementary Figure S8 reports neighbourhood and embedding metrics used to compare local batch mixing and target separation across correction outputs.

## Supplementary Tables

**Supplementary Table S1.** Public-server input limits and pre-run validation checks.

| Check | Server action | Rule shown to users |
|---|---|---|
| Feature-table format | Block if invalid | Users must upload a processed microbiome feature table in CSV format. Each row must be a sample and each column must be a microbiome feature, such as an OTU, ASV, taxon or functional feature. Compatible inputs include 16S-derived OTU/ASV tables and shotgun metagenomic taxonomic or functional abundance/count profiles after upstream profiling. Raw sequencing files, including FASTQ files, are not accepted. |
| Feature-table size | Block if exceeded | The public server accepts feature tables with at most 500 samples, 1 000 features, and 10 MB per CSV file. Larger files or matrices are rejected before correction starts. |
| Feature-table values | Block if invalid | The numeric feature table must contain numbers only. Blank cells, NA, NaN, Inf and text values are rejected before correction starts. |
| Empty sample rows | Block if present | A sample with zero values for all features is rejected before correction starts. |
| Metadata file | Block if invalid | Users must upload a metadata CSV file describing the same samples as the feature table and in the same row order. The metadata CSV file must be 10 MB or smaller. |
| Required metadata columns | Block if missing | The metadata must contain one batch column and one binary target/phenotype column. The batch column represents the technical grouping to correct. The target/phenotype column represents the biological or experimental group to preserve or compare. |
| Metadata-column limit | Block if exceeded | The metadata file can contain up to five columns total, including the batch column, target/phenotype column, and any optional covariates. |
| Metadata entries | Block if invalid | Selected metadata columns used in the analysis cannot contain missing labels or blank entries. |
| Metadata-column selection | Block if invalid | The same metadata column cannot be selected as both batch and target/phenotype. |
| Empty feature columns | Warning | A feature that is zero in every sample triggers a warning because it contains no information for comparing samples. The run can continue. |
| Outlier screening | Warning | The total abundance or count is calculated for each sample, and samples with unusually high or low totals are flagged using a 5.0× median absolute deviation (MAD) rule. This MAD-based screening follows robust QC practice, while the 5.0× threshold is used as a conservative advisory cutoff (McCarthy et al., 2017; Wolf et al., 2018). Flagged samples are not removed automatically and do not stop the run. The warning asks users to review the input table before correction. |
| Batch-target association screening | Warning | The association between the selected batch and target/phenotype columns is summarized using Cramér's $V$ from the batch-by-target contingency table (Cramér, 1946; Lee, 2016). A warning is displayed when Cramér's $V \geq 0.6$, following the "strong association" range summarized by Lee (Lee, 2016). |

**Supplementary Table S2. Supported correction methods and practical guidance.**

| Method | Practical grouping | Practical guidance |
|---|---|---|
| ConQuR (Ling et al., 2022) | Microbiome-oriented | Useful when quantile-based adjustment of microbiome profiles is desired. Covariate/target settings should be chosen to preserve biological covariates where supported. |
| MMUPHin (Ma et al., 2022) | Microbiome-oriented | Useful for microbiome population-structure and cross-study adjustment. Users should inspect both residual batch association and target preservation after correction. |
| PLSDA-batch (Wang and Lê Cao, 2023) | Microbiome-oriented | Component and tuning choices can affect the balance between batch attenuation and target separation. |
| DEBIAS-M (Austin et al., 2025) | Microbiome-oriented | Useful for processing-bias correction and cross-study generalization. Runtime and memory can be higher than simple linear methods. |
| MetaDICT (Yuan and Wang, 2025) | Microbiome-oriented | Useful when dictionary-learning-based integration is appropriate. Iteration and model-size parameters can influence runtime. |
| ComBat (Johnson et al., 2007) | General-purpose / expression-derived | Empirical-Bayes adjustment is fast and widely used, but users should check that transformed inputs and covariate design match assumptions. |
| limma (Ritchie et al., 2015) | General-purpose / expression-derived | Useful as a fast linear-model baseline. Design metadata columns should be specified carefully to avoid removing target-associated signal. |
| ComBat-seq (Zhang et al., 2020) | General-purpose / count-oriented | Appropriate for count data rather than CLR-transformed values. Users should inspect downstream relative-abundance and compositional diagnostics. |
| FAbatch (Hornung et al., 2016) | General-purpose / expression-derived | Useful when latent-factor adjustment is plausible. Overcorrection should be assessed using target-preservation diagnostics. |
| RUV-III-NB (Salim et al., 2022) | General-purpose / count-oriented | Requires attention to unwanted-factor settings and data assumptions. Diagnostics should be used to verify that biological structure is retained. |
| FSQN (Franks et al., 2018) | General-purpose / expression-derived | Fast baseline method for distributional normalization. Users should verify whether its expression-derived assumptions are suitable for their microbiome table. |
| BMC (Sims et al., 2008) | General-purpose / expression-derived | Useful as a computationally inexpensive baseline. Because it is simple, users should examine whether residual batch effects remain. |

**Supplementary Table S3.** Reference runtime and peak memory for the bundled example dataset used in the manuscript case study.

| Method | Reference elapsed time for benchmarked input (s) | Peak memory (MiB) |
|---|---:|---:|
| ConQuR | 26.57 | 377.34 |
| MMUPHin | 4.03 | 147.92 |
| PLSDA-batch | 20.47 | 267.48 |
| DEBIAS-M | 39.40 | 1096.79 |
| MetaDICT | 45.14 | 344.40 |
| ComBat | 12.42 | 561.25 |
| limma | 1.62 | 76.21 |
| ComBat-seq | 13.27 | 541.16 |
| FAbatch | 15.18 | 617.49 |
| RUV-III-NB | 17.73 | 326.01 |
| FSQN | 0.88 | 74.07 |
| BMC | 3.67 | 208.92 |

**Supplementary Table S4.** Main output and reproducibility records in the downloadable bundle.

| Output record | Purpose |
| --- | --- |
| Corrected matrices | Method-specific corrected sample-by-feature tables for downstream analyses. |
| Diagnostic figures | Pre- and post-correction ordination, dissimilarity, variance-partitioning, and neighbourhood-metric figures. |
| Statistical summaries | ANOSIM, PERMANOVA, ANOVA, pRDA, PVCA, and neighbourhood-metric summaries generated by the selected workflow. |
| `output_summary.json` | Describes generated files and their purpose so users can locate method-specific corrected matrices, diagnostic figures, statistical summaries, logs and reproducibility records. |
| `runtime_summary.json` | Records elapsed runtime and run-level performance metadata. |
| `parameter_manifest.json` | Records selected correction methods and method-specific parameters. |
| `session_config.json` | Records session metadata mapping and analysis settings, including batch/target mapping and reference-batch or control-label choices where used. |
| `execution_commands.sh` | Lists the server-side preprocessing command and executed correction-method commands, including method-specific flags where available; this script documents the command sequence used in the session and complements the reproducibility bundle. |
| `validation_report.json` | Records upload and pre-run validation checks, including input-size checks and advisory warnings. |
| Session metadata and logs | Documents session state, execution messages and run status for reproducibility and troubleshooting. |

**Supplementary Table S5. Aitchison distance-based batch-association statistics.** ANOSIM and PERMANOVA use 999 permutations unless otherwise noted.

| Method | ANOSIM $R$ | ANOSIM $p$ | PERMANOVA $R^2$ | PERMANOVA $p$ |
|---|---|---|---|---|
| Before correction | 0.507 | 0.001 | 0.323 | 0.001 |
| ConQuR | 0.017 | 0.270 | 0.061 | 0.218 |
| MMUPHin | 0.166 | 0.001 | 0.115 | 0.001 |
| PLSDA-batch | 0.202 | 0.001 | 0.143 | 0.001 |
| DEBIAS-M | 0.201 | 0.001 | 0.130 | 0.001 |
| MetaDICT | 0.221 | 0.001 | 0.131 | 0.001 |
| ComBat | 0.215 | 0.001 | 0.160 | 0.001 |
| limma | -0.112 | 1.000 | 0.007 | 1.000 |
| ComBat-seq | 0.275 | 0.001 | 0.177 | 0.001 |
| FAbatch | 0.361 | 0.001 | 0.242 | 0.001 |
| RUV-III-NB | 0.509 | 0.001 | 0.324 | 0.001 |
| FSQN | 0.338 | 0.001 | 0.231 | 0.001 |
| BMC | -0.152 | 1.000 | 0.000 | 1.000 |

**Supplementary Table S6. Bray–Curtis distance-based batch-association statistics.** ANOSIM and PERMANOVA use 999 permutations unless otherwise noted.

| Method | ANOSIM $R$ | ANOSIM $p$ | PERMANOVA $R^2$ | PERMANOVA $p$ |
|---|---|---|---|---|
| Before correction | 0.637 | 0.001 | 0.401 | 0.001 |
| ConQuR | -0.050 | 0.973 | 0.046 | 0.724 |
| MMUPHin | -0.010 | 0.601 | 0.116 | 0.002 |
| PLSDA-batch | 0.377 | 0.001 | 0.098 | 0.012 |
| DEBIAS-M | -0.052 | 0.983 | 0.127 | 0.001 |
| MetaDICT | 0.039 | 0.107 | 0.127 | 0.001 |
| ComBat | 0.047 | 0.072 | 0.154 | 0.001 |
| limma | -0.002 | 0.468 | 0.064 | 0.161 |
| ComBat-seq | 0.018 | 0.258 | 0.205 | 0.001 |
| FAbatch | 0.300 | 0.001 | 0.268 | 0.001 |
| RUV-III-NB | 0.639 | 0.001 | 0.402 | 0.001 |
| FSQN | 0.463 | 0.001 | 0.308 | 0.001 |
| BMC | 0.001 | 0.457 | 0.077 | 0.040 |

**Supplementary Table S7. Feature-wise ANOVA and pRDA summaries.** pRDA components are reported as fractions under the same settings used by the server.

| Method | ANOVA batch | ANOVA target | pRDA batch | pRDA target | pRDA intersection |
|---|---|---|---|---|---|
| Before correction | 0.273 | 0.070 | 0.267 | 0.120 | 0.017 |
| ConQuR | 0.033 | 0.083 | 0.000 | 0.159 | 0.007 |
| MMUPHin | 0.094 | 0.071 | 0.054 | 0.125 | 0.011 |
| PLSDA-batch | 0.115 | 0.065 | 0.095 | 0.161 | 0.000 |
| DEBIAS-M | 0.125 | 0.067 | 0.078 | 0.128 | 0.002 |
| MetaDICT | 0.079 | 0.060 | 0.083 | 0.121 | 0.000 |
| ComBat | 0.061 | 0.065 | 0.102 | 0.122 | 0.010 |
| limma | 0.004 | 0.067 | 0.000 | 0.117 | 0.000 |
| ComBat-seq | 0.131 | 0.069 | 0.123 | 0.132 | 0.007 |
| FAbatch | 0.225 | 0.085 | 0.205 | 0.233 | 0.000 |
| RUV-III-NB | 0.271 | 0.070 | 0.268 | 0.120 | 0.017 |
| FSQN | 0.240 | 0.050 | 0.187 | 0.077 | 0.000 |
| BMC | 0.000 | 0.062 | 0.000 | 0.133 | 0.000 |

**Supplementary Table S8. PVCA variance-partitioning summaries.** PVCA components are reported as fractions under the same settings used by the server.

| Method | PVCA batch | PVCA target | PVCA intersection | PVCA residual |
|---|---|---|---|---|
| Before correction | 0.383 | 0.275 | 0.036 | 0.305 |
| ConQuR | 0.017 | 0.392 | 0.000 | 0.592 |
| MMUPHin | 0.075 | 0.305 | 0.033 | 0.587 |
| PLSDA-batch | 0.096 | 0.344 | 0.093 | 0.467 |
| DEBIAS-M | 0.120 | 0.299 | 0.033 | 0.548 |
| MetaDICT | 0.135 | 0.276 | 0.030 | 0.559 |
| ComBat | 0.183 | 0.302 | 0.009 | 0.506 |
| limma | 0.000 | 0.339 | 0.000 | 0.661 |
| ComBat-seq | 0.189 | 0.312 | 0.022 | 0.477 |
| FAbatch | 0.328 | 0.404 | 0.016 | 0.252 |
| RUV-III-NB | 0.384 | 0.276 | 0.036 | 0.304 |
| FSQN | 0.300 | 0.243 | 0.012 | 0.445 |
| BMC | 0.000 | 0.294 | 0.000 | 0.706 |

**Supplementary Table S9. Neighbourhood and target-preservation summaries.** Alignment and entropy summarize batch mixing; silhouette summarizes target-label separation in the UMAP embedding.

| Method | Alignment | Entropy | Silhouette |
|---|---|---|---|
| Before correction | 0.379 | 0.870 | 0.608 |
| ConQuR | 0.745 | 0.966 | 0.779 |
| MMUPHin | 0.613 | 0.962 | 0.665 |
| PLSDA-batch | 0.528 | 0.938 | 0.674 |
| DEBIAS-M | 0.547 | 0.896 | 0.640 |
| MetaDICT | 0.585 | 0.962 | 0.632 |
| ComBat | 0.600 | 0.961 | 0.670 |
| limma | 0.764 | 0.966 | 0.752 |
| ComBat-seq | 0.516 | 0.896 | 0.628 |
| FAbatch | 0.407 | 0.871 | 0.644 |
| RUV-III-NB | 0.381 | 0.875 | 0.610 |
| FSQN | 0.420 | 0.907 | 0.580 |
| BMC | 0.756 | 0.970 | 0.708 |

## Supplementary Figures

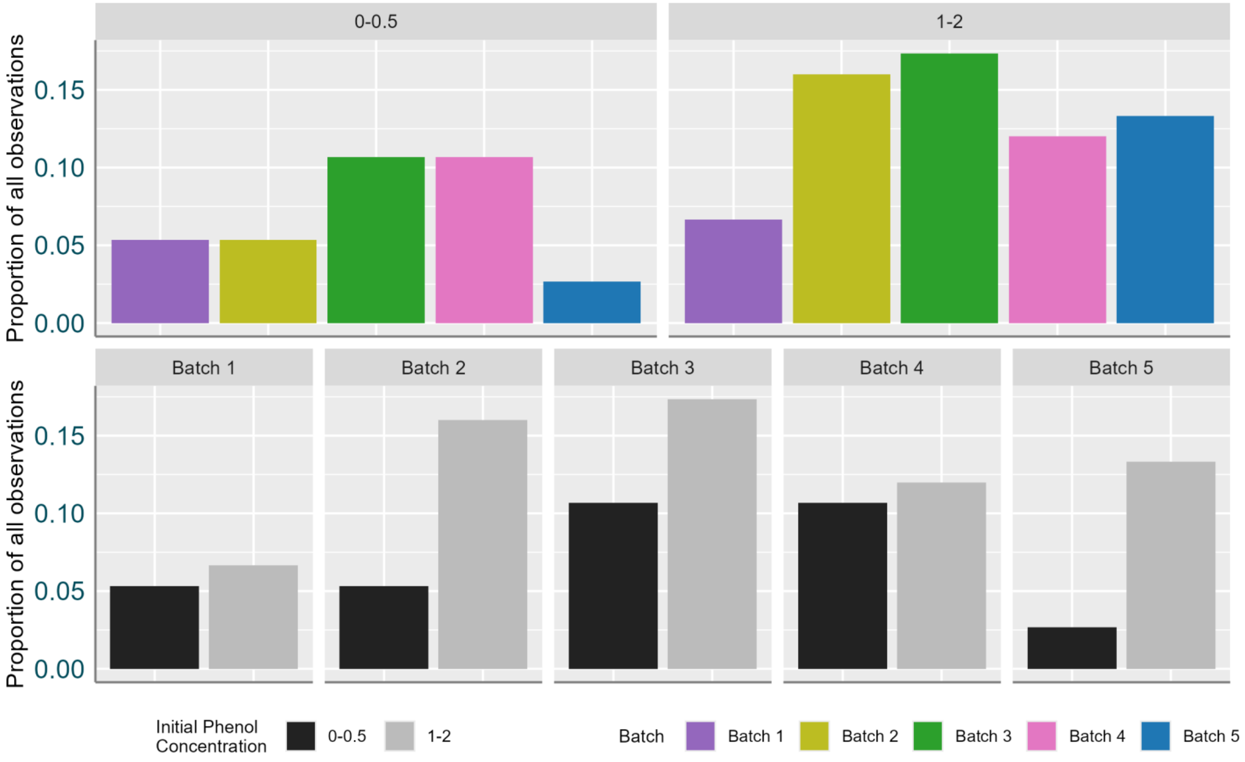


**Supplementary Figure S1. Sample composition by batch and target group.** Proportion of samples in each batch stratified by the two target (phenotypic) cohorts.

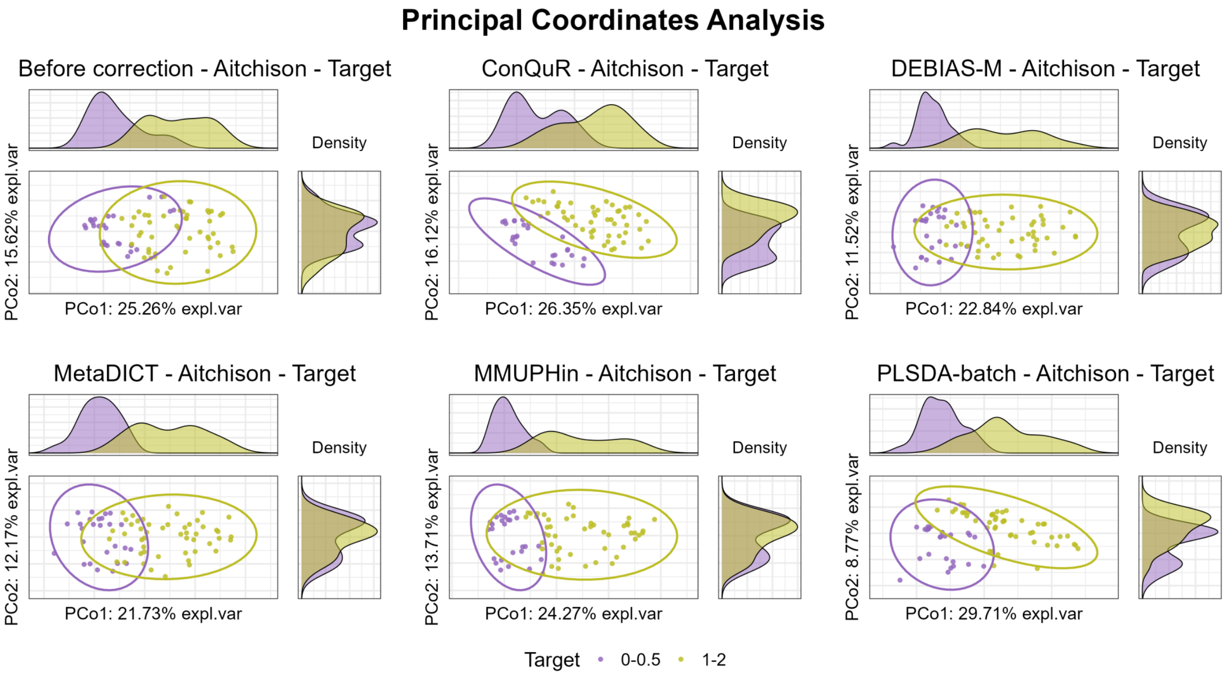


**Supplementary Figure S2. Aitchison-distance PCoA coloured by target group.** PCoA ordinations in Aitchison space, coloured by the two target cohorts, shown before correction and after each of the five representative case-study methods.

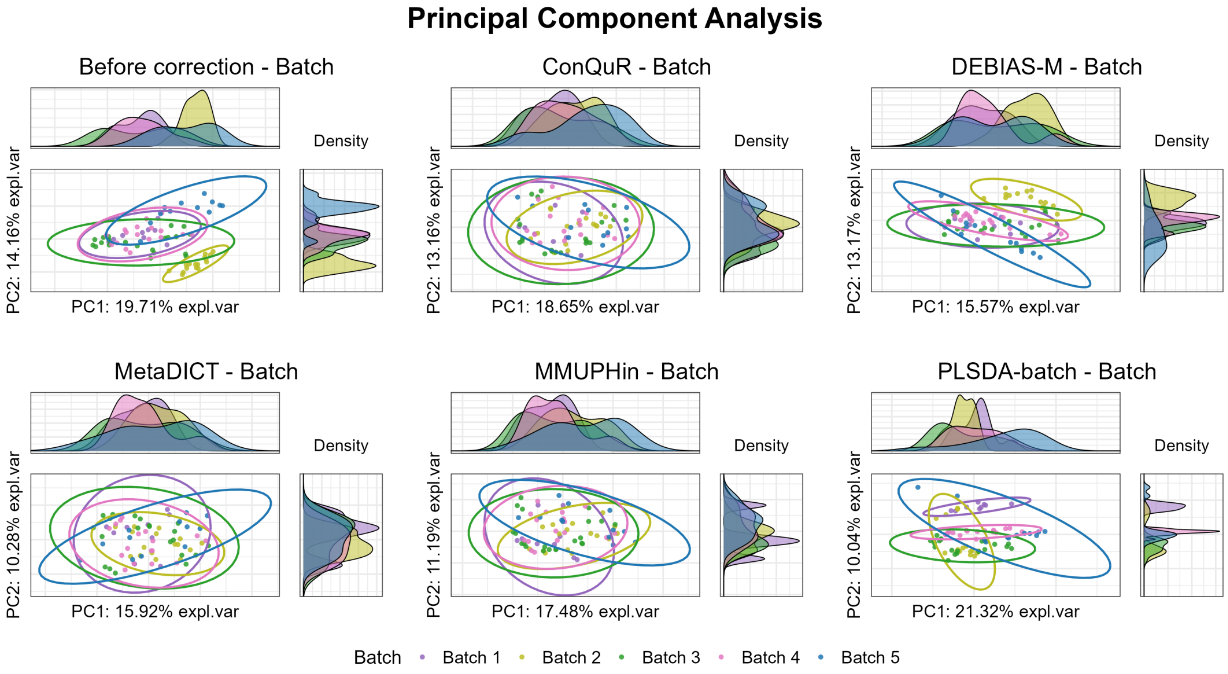


**Supplementary Figure S3. PCA coloured by batch.** PCA embeddings coloured by batch, shown before correction and after each batch-correction method.

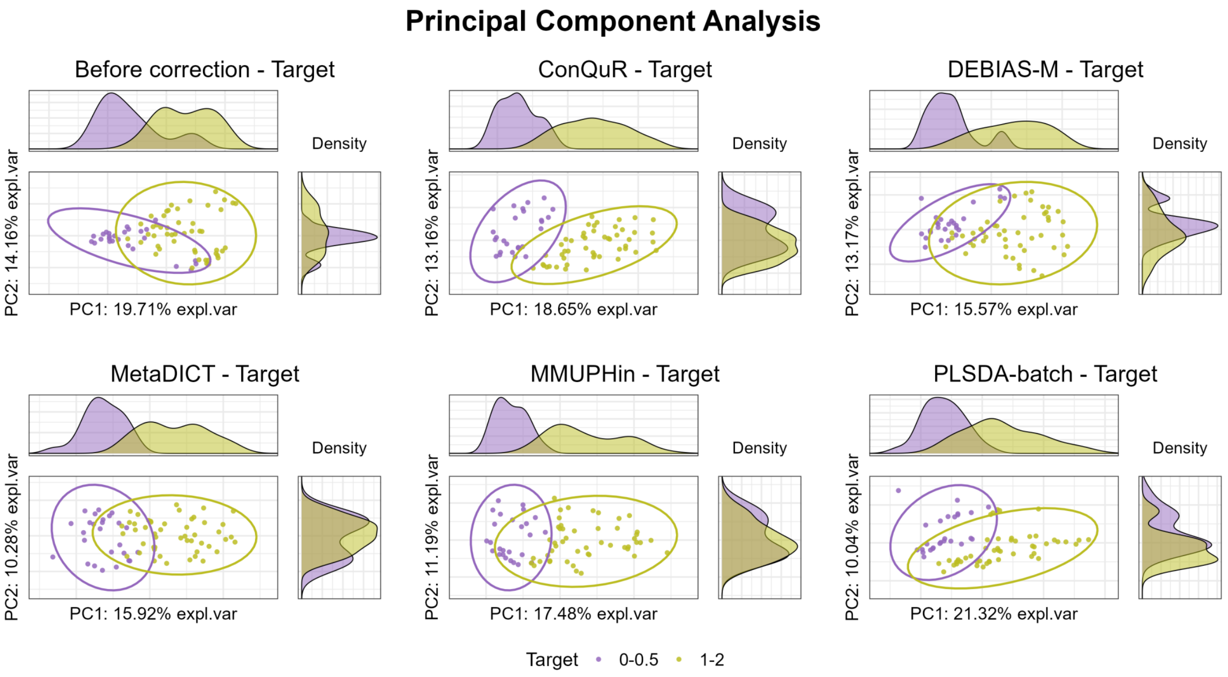


**Supplementary Figure S4. PCA coloured by target group.** PCA embeddings coloured by the two target cohorts, shown before correction and after each batch-correction method.

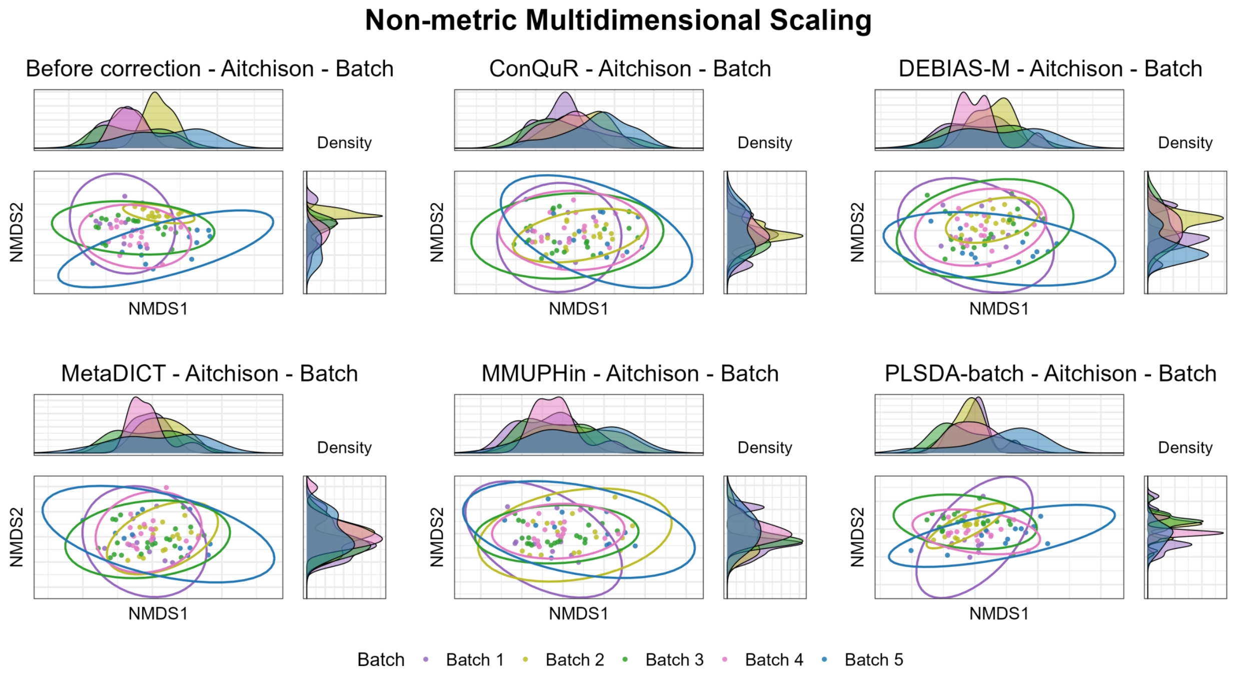


**Supplementary Figure S5. Aitchison-distance NMDS coloured by batch.** NMDS ordinations in Aitchison space coloured by batch, shown before correction and after each batch-correction method.

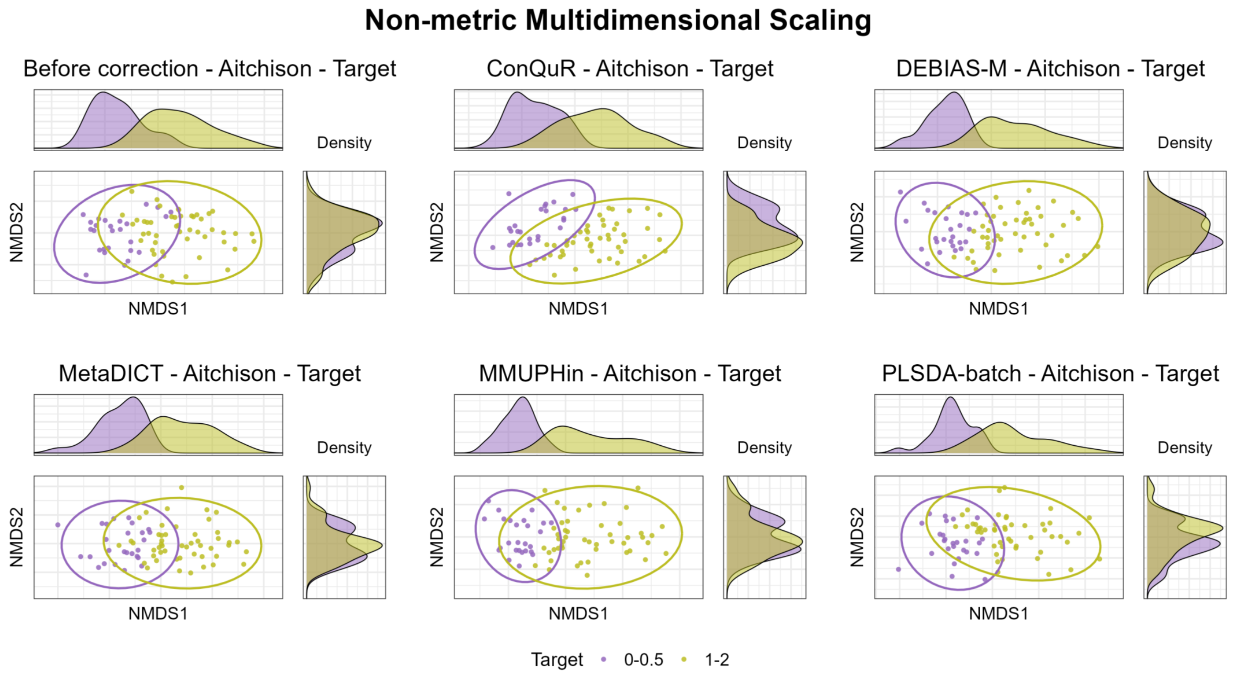


**Supplementary Figure S6. Aitchison-distance NMDS coloured by target group.** NMDS ordinations in Aitchison space coloured by the two target cohorts, shown before correction and after each batch-correction method.

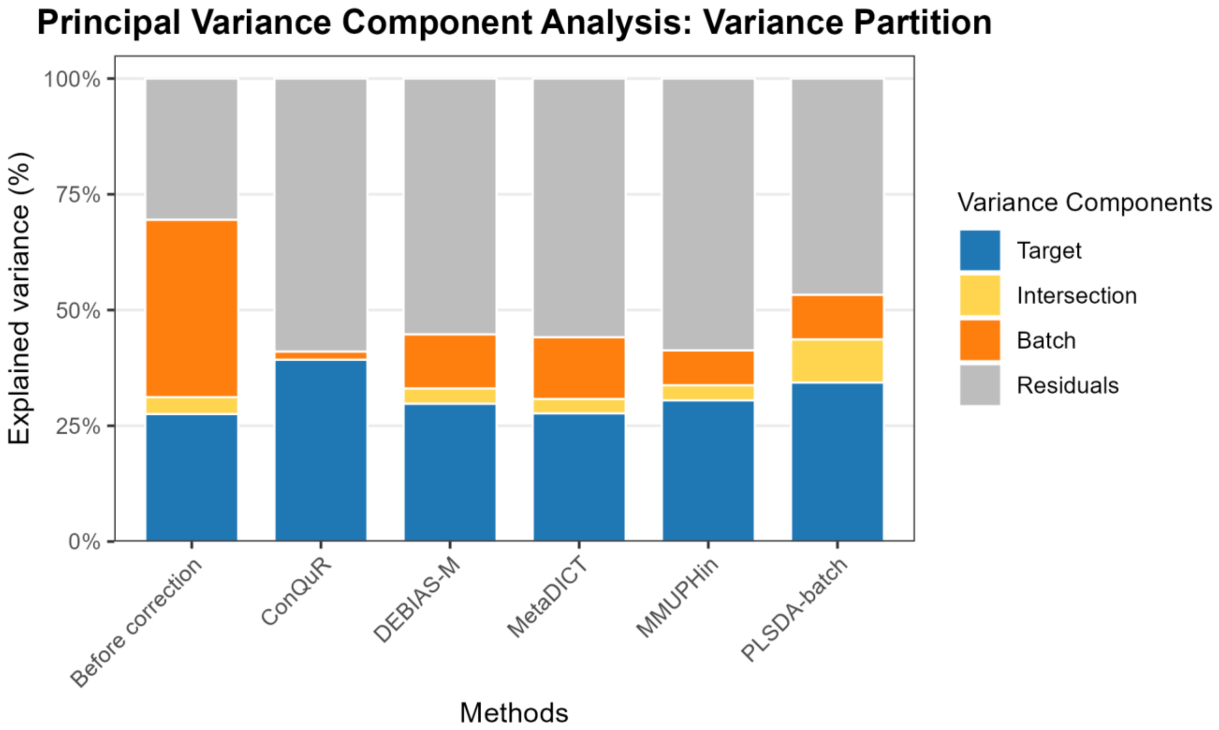


**Supplementary Figure S7. PVCA variance partition across methods.** Principal variance component analysis (PVCA) showing the fraction of variance attributable to target, batch, their overlap, and residual components for each representative case-study method.

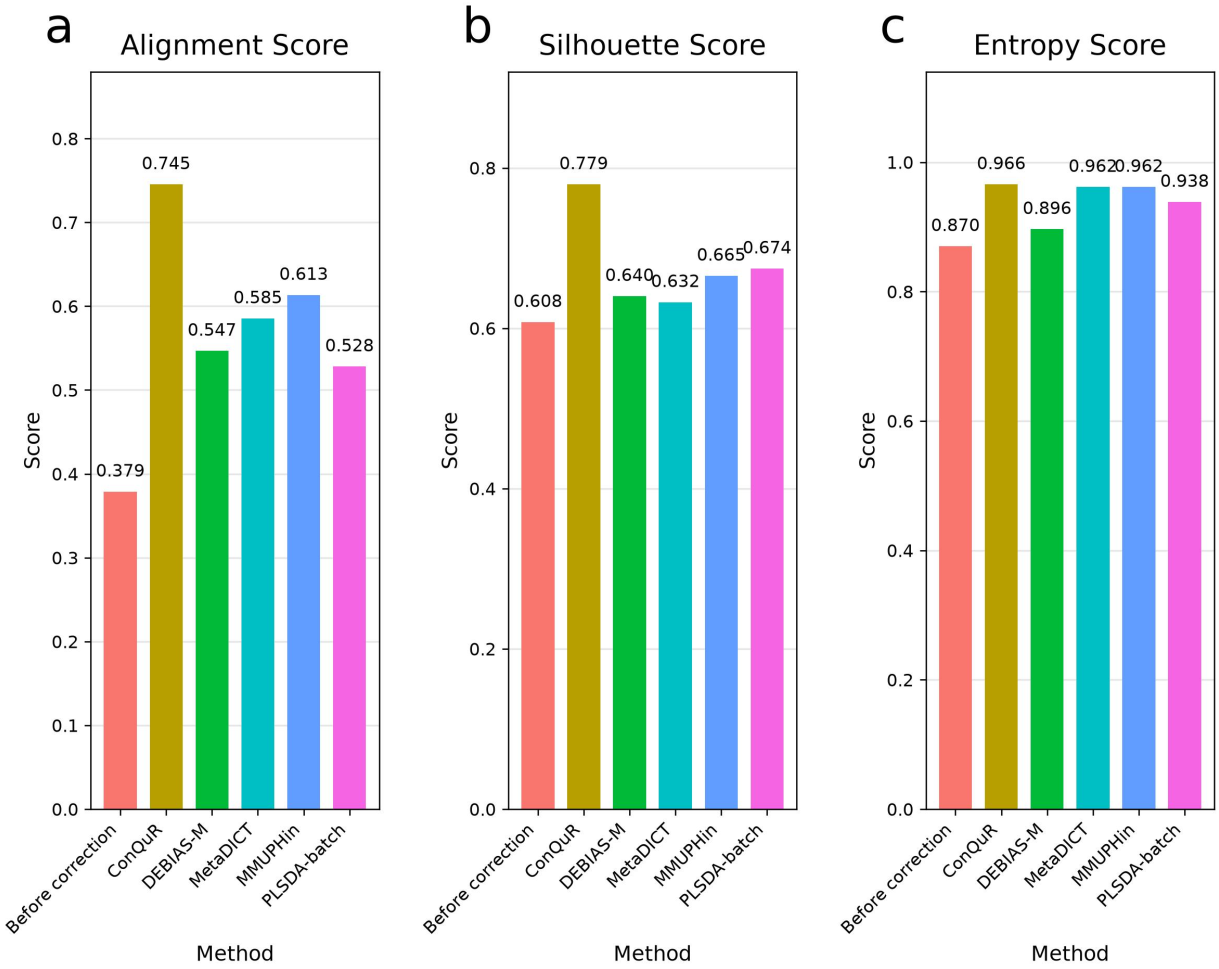


**Supplementary Figure S8. Batch-correction evaluation metrics across methods.** Comparison of alignment, silhouette, and entropy-based metrics across the five representative case-study methods, summarizing cross-batch mixing and target separation.

## Supplementary References


Aitchison J. The statistical analysis of compositional data. *J R Stat Soc Ser B Methodol* 1982;44:139–77.

Anderson MJ. A new method for non-parametric multivariate analysis of variance. *Austral Ecol* 2001;26:32–46.

Austin GI et al. Processing-bias correction with DEBIAS-M improves cross-study generalization of microbiome-based prediction models. *Nat Microbiol* 2025;10:897–911.

Bray JR, Curtis JT. An ordination of the upland forest communities of southern Wisconsin. *Ecol Monogr* 1957;27:325–49.

Butler A et al. Integrating single-cell transcriptomic data across different conditions, technologies, and species. *Nat Biotechnol* 2018;36:411–20.

Clarke KR. Non-parametric multivariate analyses of changes in community structure. *Aust J Ecol* 1993;18:117–43.

Cramér H. *Mathematical Methods of Statistics*. Princeton: Princeton University Press; 1946.

Franks JM, Cai G, Whitfield ML. Feature specific quantile normalization enables cross-platform classification of molecular subtypes using gene expression data. *Bioinformatics* 2018;34:1868–74.

Haghverdi L et al. Batch effects in single-cell RNA-sequencing data are corrected by matching mutual nearest neighbors. *Nat Biotechnol* 2018;36:421–7.

Hornung R, Boulesteix A-L, Causeur D. Combining location-and-scale batch effect adjustment with data cleaning by latent factor adjustment. *BMC Bioinformatics* 2016;17:27.

Johnson WE, Li C, Rabinovic A. Adjusting batch effects in microarray expression data using empirical Bayes methods. *Biostatistics* 2007;8:118–27.

Lee DK. Alternatives to P value: confidence interval and effect size. *Korean J Anesthesiol* 2016;69:555–62.

Li L et al. An in vitro model maintaining taxon-specific functional activities of the gut microbiome. *Nat Commun* 2019;10:4146.

Ling W et al. Batch effects removal for microbiome data via conditional quantile regression. *Nat Commun* 2022;13:5418.

Luecken MD et al. Benchmarking atlas-level data integration in single-cell genomics. *Nat Methods* 2022;19:41–50.

Ma S et al. Population structure discovery in meta-analyzed microbial communities and inflammatory bowel disease using MMUPHin. *Genome Biol* 2022;23:208.

McCarthy DJ et al. Scater: pre-processing, quality control, normalization and visualization of single-cell RNA-seq data in R. *Bioinformatics* 2017;33:1179–86.

McInnes L et al. UMAP: Uniform Manifold Approximation and Projection. *J Open Source Softw* 2018;3:861.

Ritchie ME et al. limma powers differential expression analyses for RNA-sequencing and microarray studies. *Nucleic Acids Res* 2015;43:e47.

Rousseeuw PJ. Silhouettes: a graphical aid to the interpretation and validation of cluster analysis. *J Comput Appl Math* 1987;20:53–65.

Salim A et al. RUV-III-NB: normalization of single cell RNA-seq data. *Nucleic Acids Res* 2022;50:e96.

Sims AH et al. The removal of multiplicative, systematic bias allows integration of breast cancer gene expression datasets: improving meta-analysis and prediction of prognosis. *BMC Med Genomics* 2008;1:42.

Wang Y, Lê Cao K-A. PLSDA-batch: a multivariate framework to correct for batch effects in microbiome data. *Brief Bioinform* 2023;24:bbac622.

Wolf FA, Angerer P, Theis FJ. SCANPY: large-scale single-cell gene expression data analysis. *Genome Biol* 2018;19:15.

Yuan B, Wang S. Microbiome data integration via shared dictionary learning. *Nat Commun* 2025;16:8147.

Zhang Y, Parmigiani G, Johnson WE. ComBat-seq: batch effect adjustment for RNA-seq count data. *NAR Genom Bioinform* 2020;2:lqaa078.
